# Alkaloid extracts from *Combretum zeyheri* inhibit the growth of *Mycobacterium smegmatis*

**DOI:** 10.1186/s12906-017-1636-0

**Published:** 2017-02-23

**Authors:** Tafadzwa Nyambuya, Ruvimbo Mautsa, Stanley Mukanganyama

**Affiliations:** 1 School of Pharmacy, College of Health Sciences, University of Zimbabwe, Mt. Pleasant, Harare Zimbabwe; 2 Department of Biochemistry, University of Zimbabwe, P.O. Box MP 167, Mt. Pleasant, Harare Zimbabwe

**Keywords:** Combretum species, *Mycobacterium smegmatis*, Drug efflux, Drug susceptibility test, Alkaloid extracts

## Abstract

**Background:**

Current tuberculosis regimens have failed to combat the issue of drug resistance and ethno medicines may represent a possible source of antimycobacterial agents. Combretum species are well known in African traditional medicines and used for various ailments including pneumonia, venereal diseases like syphilis, mental problems, relief of sore throats and colds, fever, and chest coughs associated with tuberculosis. Alkaloids function as either hydrogen-acceptor or hydrogen-donor in hydrogen bonding critical for the interaction between targets thus, potentiating effects of curative agents on diseases. Alkaloid extracts from leaves of *Combretum zeyheri*, *Combretum platypetalum*, *Combretum molle* and *Combretum apiculatum*, were assessed for antimycobacterial activity to establish rationale for their use in traditional medicines for various ailments including pneumonia, relief of sore throats and colds, fever, and chest coughs associated with tuberculosis.

**Methods:**

Alkaloids were extracted from the leaves of *Combretum zeyheri*, *Combretum platypetalum*, *Combretum molle* and *Combretum apiculatum*. The broth microdilution method was used for the screening of growth inhibitory activity. The standard drug rifampicin was used as the positive control. Alkaloid extracts from the most potent plant species, *Combretum zeyheri* were further investigated for time-kill dependency effects on drug transport in *Mycobacterium smegmatis*.

**Results:**

Using the broth microdilution susceptibility method, *C. zeyheri* alkaloid extract, was found to have the most antimycobacterial effects with an MIC value of 125 μg/ml whilst MICs for *C. molle* and *C. platypetalum* were above 1000 μg/ml. An MBC value of 250 μg/ml was observed with alkaloid extracts from *Combretum zeyheri* whilst the remaining three *Combretum* species showed no bactericidal activity. It was also shown that *C. zeyheri* had potential efflux pump inhibitory activity. Determination of the time-kill kinetics of extracts from *C. zeyheri* showed not only a concentration-dependent activity but time-dependent bactericidal effect as well.

**Conclusions:**

Alkaloid extracts from the leaves of *C. zeyheri* have potential as a source of lead compounds that may be developed further into antimycobacterial compounds. The mechanism of action of may be due to inhibition of transport across the cell membrane. Further work needs to be done to isolate the active components in these extracts.

## Background

Tuberculosis (TB) is a bacterial infection caused by a bacterium called *Mycobacterium tuberculosis*. Unique about mycobacterium, is the cell wall structure which contains mycolic acids intercalated to peptidoglycan – associated polysaccharides [1]. There are various strains of mycobacterium and transmission is through the lymph nodes and the blood stream to any organ of the body but mainly affects the lungs causing pulmonary tuberculosis. It may disseminate to other body parts causing extra-pulmonary tuberculosis [2]. The bacterium generally exists in its inactive form and only a few people infected will develop the active form of the disease [3]. Approximately 8 million TB cases emerge every year whilst at most 5000 people die from the disease everyday worldwide [4].

In most developing countries there is a relationship between HIV/AIDS and tuberculosis. According to the World Health Organisation (WHO) one-third of patients living with HIV are infected with TB [5]. Patients living with HIV are 35 times more likely to become infected with TB than people without HIV [6]. The treatment regimens consist of a combination of 5 first line medicines; rifampicin, isoniazid, pyrazinamide, ethambutol and streptomycin (HREZS) [7]. The initial intensive phase of includes rifampicin, isoniazid, pyrazinamide and, ethambutol daily for 2 months and a continuation phase of rifampicin and isoniazid for a further 4 months, either daily or 3 times a week [8]. Resistance of mycobacterium to conventional drugs is mainly due to inadequate exposure of the TB causing organism to anti-tuberculosis drugs [9]. The ability of mycobacterium strains to increase activity of their efflux pumps preventing drugs from reaching intended targets is amongst the major reasons of multi-drug resistant tuberculosis (MDR-TB) [10]. Multi-drug resistance TB occurs when mycobacterium do not respond to at least isoniazid and rifampicin, which are part of the 2 first line or standard anti-TB drugs. Extensive-drug resistance occurs when mycobacterium becomes resistant to most of the available drugs including most second line anti-TB drugs [5].

There is a global increase in the use of herbal medicines and developing countries tend to depend on these plants more than other populations primarily because of the limitations in modern healthcare facilities in addition to cultural preferences [11]. To date, phytomedicines have shown great potential in treating intractable infectious diseases for example TB [4]. Approximately 80% of the population in developing countries depend on traditional medicines for their primary health care needs [12]. In acknowledgement of this fact, the WHO declared a resolution on promoting the role of traditional medicine in health systems, a strategy for the African region. For most plants safety and efficacy profiles have not yet been identified and this has posed a major challenge in trying to merge traditional medicines and modern medicines in order to improve the health care system. There is a need, therefore, to evaluate and justify their respective pharmacological activities.

The family *Combretaceae* consists of approximately 20 genera and 600 species. This plant occurs mainly in tropical and subtropical areas, like Africa and Brazil [13]. The *Combretaceae* family of plants has been widely used as traditional medicines [14]. Use of these plants to treat scorpion and snake bites, and mental problems and their use for the relief of sore throats and colds, fever, chest coughs associated with tuberculosis, pneumonia and venereal diseases like syphilis is common in most African communities [15]. Studies on the genus have shown presence of several phytochemical constituents including alkaloids, saponins, tannins and cardiac glycosides [16, 17]. To date there are over 27 000 alkaloid-based compounds in the Dictionary of Natural Products (DNPs) [18]. Alkaloid molecules can act, depending on a type of amine functionality present in alkaloids, as either hydrogen- acceptor or hydrogen-donor for hydrogen bonding. This bonding is critically important for the interaction (binding) between targets, which may be enzymes, proteins and receptors for drugs, thereby, potentiating the drug effects in a pathology condition [19].

There is need for in vitro-screening of phytomedicines so that there is validation of their traditional use and for providing leads in the discovery of new active chemical principles [3, 20]. The highly infectious nature of *Mycobacterium tuberculosis* restricts its use for large scale screening of probable drug candidates [21]. *Mycobacterium smegmatis* is a fast growing and non-pathogenic strain compared to the disease-causing strain *M. tuberculosis. M. smegmatis* has been found to display a similar drug sensitivity profile similar to *M. tuberculosis* [21] and, therefore, this organism can be used as a primary screen to shortlist compounds with antimycobacterial activity. The objectives of this study, therefore, was to evaluate the effects of alkaloid extracts from selected Combretum species on a model mycobacterium species, *M. smegmatis.*

## Methods

### Reagents

The following chemicals and equipment were used and these are: Phillips two-speed electric blender, 10% Ethanolic acetic acid, ammonia solution, Middlebrook 7H9 media, casein hydrosylate, agar, an incubator, dimethyl sulphoxide (DMSO), dichloromethane (DCM), rifampicin, ciprofloxacin, 96-well microtitre plates, a Biokinetics Reader EL350, biosafety hazard safety cabinet level 2. All the chemicals used were obtained from Sigma Aldrich (Darmstadt, Germany)

### Plant collection and preparation of extracts

*Combretum zeyheri* and *Combretum platypetalum* leaves were collected in Norton, Zimbabwe, geographical location 17.8833 ° S, 30.7000 ° E, 1364 m above sea-level. *Combretum molle and Combretum apiculatum* leaves were collected in Centenary (16.8°S, 31.1167°E, and 1156 m above sea level), Mashonaland Central Province, Zimbabwe in the summer period (January-February, 2013). The plants identity was authenticated and classified by Mr. Christopher Chapano, a taxonomist at the National Herbarium and Botanic Gardens (Harare, Zimbabwe). The samples were allocated a voucher specimen number N6E7, N9E7, C1E7 and C2E7 for *C. zeyheri, C. platypetalum, C. apiculatum and C. molle* respectively and herbarium samples were kept at the National Botanic and Herbarium Garden and the Department of Biochemistry, University of Zimbabwe. The dried plant leaves were ground using a two speed blender (Cole Parmer instruments company, Vernon Hills, USA). Alkaloid phytoconstituents were extracted from the plants using a polar solvent, 20 ml of 10% ethanolic acetic acid after which the mixtures were left to stand for 4 h at room temperature a method described by Harbone [22] with modifications. Mixtures were filtered through a Whatman filter paper. The filtrate was concentrated by evaporation over a steam bath to a quarter of its original volume. To precipitate the alkaloid, concentrated ammonia solution was added in drops to the extract until it is in excess. Alkaloid precipitates were recovered by filtration using previously weighed filter paper after which 9% ammonia solution was added to wash the precipitates. The precipitates were dried in an oven at 60 °C for 30 min and reweighed [23].

### Growth of mycobacteria

*Mycobacterium smegmatis*, Mc^2^155 was obtained from Professor Daniel Steenkamp of the Department of Clinical Laboratory Studies, University of Cape Town, South Africa. A volume of 100 ml of liquid broth consisting of 0.52 g Middlebrook 7H9 media supplemented with 0.1 g casein hydrosylate was prepared using boiled distilled water, sterilised by autoclaving and used for the growth of mycobacterium at 37 °C under aerobic conditions.

### Determination of antimycobacterial activity

The alkaloid extracts from the plants were used to determine the minimal inhibition concentration (MIC) and the minimal bactericidal concentration (MBC) using the broth microdilution assay described by Martini and Eloff [24]. Extracts were initially dissolved in DMSO and, therefore, the effects of DMSO were also investigated on *M. smegmatis*. The alkaloid extracts were serially diluted with media from 1000 μg/ml up to 0.2 μg/ml using the broth. Aliquots of 100 μl were placed into 96-well microtitre plates in duplicate. Rifampicin was used as the positive control. The plates were sealed with parafilm™ paper and incubated in a container containing wet paper towel over night at 37 °C in an incubating shaker (Jeio Tech, Korea). Optical densities of the wells were read using a microplate reader (Tecan Genios-Pro microplate reader, GrÖdig, Austria) at 590 nm. A solution of 3-(4, 5-dimethylthiazol-2-yl)-2, 5-diphenyltetrazolium bromide (MTT) was added and the plates were incubated for 2 h. After the incubation, 25 μL of DMSO was added to dissolve the purple formazan that would have been formed. The wells that did not show any colour change after MTT was added, indicated the concentration of the plant alkaloid extract that was able to inhibit mycobacterium growth whereas a purple colour change indicated mycobacterium growth [25]. MBCs of the alkaloids were determined on Middlebrook media 7H9 agar.

### Determination of drug efflux

Accumulation and efflux of the fluoroquinolone, ciprofloxacin was performed in order to determine the effect of plant alkaloid extract on mycobacterium efflux pumps as described by Mortimer and Piddock [26] with some modifications. Mycobacterium were grown in Middlebrook 7H9 supplemented with casein hydrosylate media in 3 separate flasks containing 400 mls media and grown over 2 days at 37 °C. The mycobacteria were harvested by centrifugation at 3000 rpm in a Hettich Rotofix 32 centrifuge (Tuttlingen, Germany) for 10 min in pre-weighed tubes and the supernatant discarded. Bacteria were then washed twice with 50 mM sodium phosphate buffer (PBS) (pH 7.2). The cells were weighed and a volume needed to make up a volume of 40 mg/ml was made in 10 mM PBS containing sodium azide. The mixture was incubated at 37 °C for 15 min. Ciprofloxacin was added to the mixture to a final concentration of 20 μg/ml. The mixture was incubated at 37 °C with shaking at 120 rpm for 1 h (Lab Companion, Jeio Tech, Seoul, South Korea). The sample was divided into two aliquots a two-third and one-third volume sample which was centrifuged at 3000 rpm for 5 min. The supernatant was discarded and the pellet was weighed. PBS was added to the one third sample to make up to a concentration of 40 mg/ml, this tube representing a sample without glucose. After discarding the supernatant and weighing the pellet PBS containing 1 M glucose was added to the two-third sample which was further sub-divided into 3 equal aliquots of 40 mg/ml containing either reserpine, DMSO, or the alkaloid plant extract from *Combretum zeyheri*.

In order to rule out the effect of the solvent DMSO, was also added in one of the tubes as it was the organic solvent used to dissolve reserpine and the alkaloid extract. The samples were mixed by vortexing before being incubated for 30 min at 37 °C with shaking at 120 rpm. After incubation, cells were washed with chilled phosphate buffer and re-centrifuged for 10 min at 4000 rpm. After the supernatant was discarded, the pellet was re-suspended in glycine hydrochloride 3.0 ml (0.1 M, pH 3.0) with agitation to ensure exposure of the cells to the lysis buffer. The samples were incubated at 37 ˚C, 120 rpm for overnight. The samples were then centrifuged for 10 min at 3000 rpm to sediment cell debris. The fluorescence of ciprofloxacin in the supernatant was determined at the excitation and emission wavelengths of 270 nm and 452 nm respectively using an RF – 1501 Shimadzu spectrofluorimeter (Shimadzu Cooperation, Tokyo, Japan). Both supernatant samples from the intact cells (representing efflux samples) and the lysed cells (representing influx samples) were quantified for ciprofloxacin using a standard curve. To rule out interference from the reserpine and the plant extracts, their fluorescence was determined at the same excitation and emission wavelength as ciprofloxacin.

### Time-kill assay

Time-kill assays were carried out with modifications from Oladosu et al., [27]. *M. smegmatis* was grown overnight at 37 °C in Middlebrook 7H9 media supplemented with casein hydrolysate. The plant alkaloid extracts were serially diluted with media from 1000 μg/ml up to 0.2 μg/ml to make ten 2-fold microdilutions for the microbroth dilution assay. Aliquots of 100 μl were plated onto 96-well microtitre plates in duplicate. Rifampicin was used as the positive control at 2-fold increasing concentrations of from 0.1 μg/ml to 50 μg/ml. The extracts and rifampicin were cultured with the inoculum at a final concentration of 1 × 10^6^ cfu/ml. In order to rule out interference from the media the plant extract was incubated with media only. The plates were sealed with parafilm™ paper and incubated in a container containing wet paper towel over night at 37̊C in an incubating shaker (Jeio Tech, Korea). At defined time intervals (0, 2, 4, 6, 8, and 24 h), the size of the bacterial population was quantified to characterise the effect of the different extract concentrations. This was done by plating 10 μl aliquots of each dilution (10 μl samples diluted up to 1 ml with media) and plated on 40 ml solid agar plates. Only 250 μg/ml, 125 μg/ml, and 63 μg/ml extract concentrations were plated. Plates were incubated for 24 h at 37 °C. The number of viable organisms were counted as cfu/plate. Surviving organisms were determined by the plate count method at sampling time and enumerated. Average duplicate (2 plates from each replicate dilution) counts were multiplied by the dilution factor to derive the concentration of cells as cfu/ml. The percentage reduction from initial microbial population for each time point was calculated to express the change (reduction or increase) of the microorganism population relative to a starting inoculum. The change was determined as follows:

$$ \%\ \mathbf{Reduction} = \left(\mathbf{initial}\ \mathbf{count}\ \hbox{--}\ \mathbf{count}\ \mathbf{at}\ \mathbf{x}\ \mathbf{interval}/\mathbf{initial}\ \mathbf{count}\right) \times \mathbf{100} $$

The number of surviving microorganisms in the extract was determined by plate-count method at sampling time and enumerated. Optical densities signifying bacterial cell density of the wells were also read using a Genios Pro microplate reader (Tecan Instruments, Grodig, Austria) at 590 nm. The results were analysed using ANOVA with graph pad ™ version 5 for Windows (Graph pad ™ Software Inc. Scan Diego, California. USA).

### Statistical analyses

Numerical data was analysed using graph pad ™ version 5 for Windows (Graph pad ™ Software Inc. Scan Diego, California. USA). Statistical analysis was carried out using One-way ANOVA where values of P < 0.05 are regarded as significant (95% confidence interval). A post-hoc analysis (Dunnet’s multiple comparison test) was used Graphpad Prism (Version 5.0, Graph pad Software Inc, San Diego, USA).

## Results

### Extraction of plant phytoconstituents and antimycobacterial activity determination

**Fig. 1:**
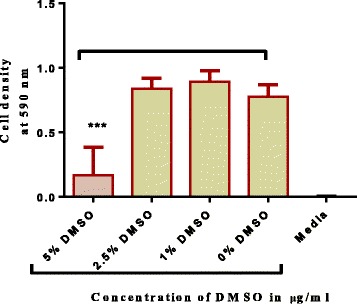
The effect of different concentrations of DMSO on *M. smegmatis. Mycobacterium smegmatis* was grown in broth and exposed to varying concentrations of DMSO in 96-well microplates. *** *P* < 0.001 in comparison with the 0% DMSO. Values are the mean ± SD for *N* = 6

**Fig. 2:**
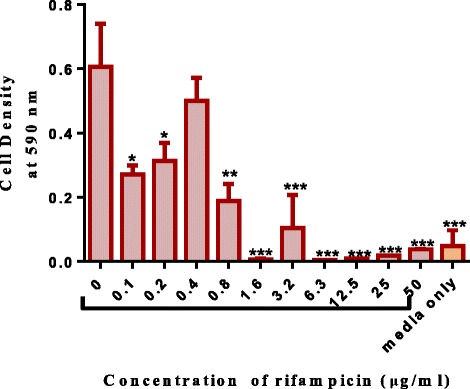
The effects of rifampicin (positive control) on the growth of mycobacterium in broth. M. smegmatis were grown and exposed to increasing concentrations of rifampicin in a 96-well microplate. Values are the mean of ± SD for n = 4 for duplicate measurements *P < 0.05 ** *P* < 0.01, *** *P* < 0.001 in comparison with the cells exposed to the zero concentration of extract

**Fig. 3:**
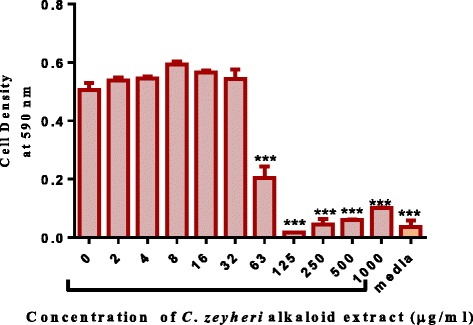
The growth inhibitory properties of alkaloid extracts from *C. zeyheri* on *Mycobacterium smegmatis*. The broth microdilution method was used to determine growth inhibitory properties of extracts using a 96-well microtitre plate. A minimum inhibitory concentration of 125 μg/ml was observed and the optical densities of the wells were read using a microplate reader (Tecan Genios-Pro microplate reader, GrÖdig, Austria) at 590 nm. Values are the mean of ± SD for *n* = 4.*** *P* < 0.001 in comparison with the cells exposed to the zero concentration of extract

**Fig. 4:**
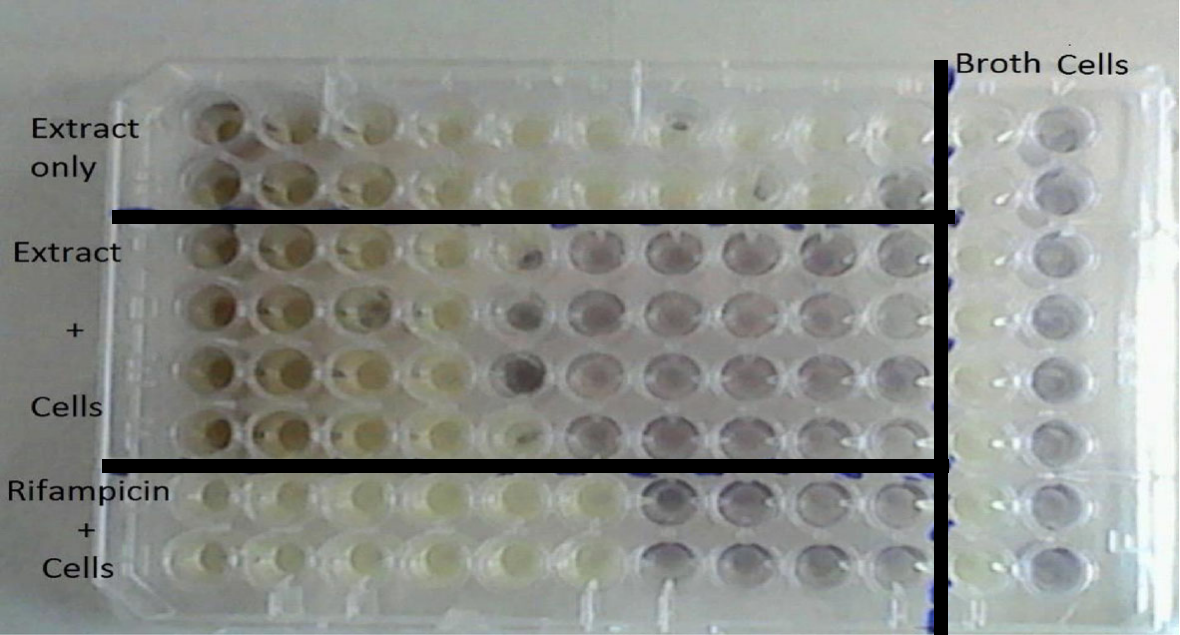
MTT assay showing the growth inhibitory effects of alkaloid extracts from *C. zeyheri* on *M. smegmatis*. Viable cells are coloured *purple* whilst metabolically inactive cells are coloured *yellow*. The intensity of the *purple* also shows levels of cell activity

**Fig. 5:**
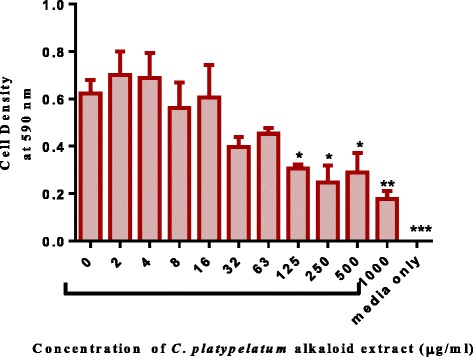
The effects of *C. platypetalum* alkaloid extracts on mycobacterium in broth. *M. smegmatis* were grown in broth and exposed to increasing concentrations of *C. platypetalum* leaf alkaloid extracts in a 96-well microplate. Values are the mean of ± SD for n = 4. *P < 0.05; ** *P* < 0.01 in comparison with the cells exposed to the zero concentration of extract

Alkaloid extracts from 4 plant species namely: *Combretum apiculatum*, *Combretum molle*, *Combretum platypetalum* and *Combretum zeyheri* were tested for antimycobacterial activity. Extraction was carried out using 10% acetic ethanolic acid, and percentage yields are shown in Table 1. The effects of the solvent was determined and the results are shown in Fig. 1. A DMSO solvent concentration of 2.5% was used for all the subsequent assays. In order to quantify the activity of alkaloid extracts from the four plant species, the broth microdilution method was used. This allowed determination of the minimum inhibitory concentration and subsequently the minimum bactericidal concentration. Varying concentrations of the plant extracts were tested and these ranged from 1000 μg/ml to 2 μg/ml attained by 2-fold dilutions. Of the four plant species extracts tested, *C. zeyheri* alkaloid extract was the most effective in inhibiting the growth of *Mycobacterium smegmatis*. Rifampicin was used as the positive control whilst the negative control was cells and media only (Fig. 2). Figure 3 shows the effects of the *C. zeyheri* alkaloids on the growth of mycobacteria. MICs of 1000 μg/ml, >1000 μg/ml and 125 μg/ml were obtained for *C. molle*, *C. platypetalum* and *C. zeyheri* respectively (Table 2). The purple formazan colour that was quantified spectrophotometrically indicated presence of viable cells showing mycobacterium growth (Fig. 4). Wells that showed no colour change after addition of MTT (yellow colour) represented concentration of the plant extracts that were able to inhibit mycobacterium growth (1000 – 125 μg/ml). *Combretum platypetalum* alkaloid extracts also showed that they had antimycobacterial effects but the these effects were lower than those of *C. zeyheri* (Fig. 5)

**Table 1:** The extractive values and percentage yields of crude 10% ethanolic acetic acid extracts

Name of plant species	Amount extracted per 50 g sample^a^	Percentage yield (%)
*C. platypetalum*	0.108	0.432
*C. apiculatum*	0.049	0.196
*C. zeyheri*	0.605	2.42
*C. molle*	0.371	1.484

The alkaloids were extracted according to the method by Harbone [23]

**Table 2:** The effects of the alkaloid –enriched leaf extracts against *Mycobacterium smegmatis* at 1000 μg/ml

Combretum species	Activity at 1000 μg/ml^a^	MIC (μg/ml)^b^
*C. platypelatum*	Moderately Active	>1000
*C. apiculutum*	Inactive	>1000
*C. molle*	Moderately active	1000
*C. zeyheri*	Active	125

^a^Activity at 1000 μg/ml, ^b^Minimum inhibitory concentration

### *Time*–*kill kinetics of alkaloid extracts from C. zeyheri* on *Mycobacterium smegmatis*

**Fig. 6:**
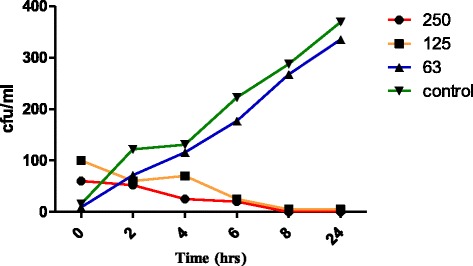
Time dependent and concentration – dependent bactericidal effect of alkaloid extracts from *C. zeyheri* on *M. smegmatis*. Cultures of *M. smegmatis* standardised to 1 x 10^6^ cfu/ml were exposed to alkaloid extracts from *C. zeyheri* at 2-fold increasing concentrations from 2 μg/ml to 1000 μg/ml over 24 h at 37 ˚C. After 2, 4, 6, 8 and 24 h of exposure samples were taken to determine number of surviving populations at 0, 63, 125, and 250 μg/ml

Alkaloid extracts from *Combretum zeyheri* showed concentration-dependent killing with a minimum inhibitory concentration of 125 μg/ml and a minimum bactericidal concentration of 250 μg/ml using the broth microdilution method (Fig. 6). Concentrations from 0-1000 μg/ml were used. In order to also determine killing capacity of the extract; a time-kill assay was carried out. For the time-dependent assay, a range of three concentration 63 μg/ml, 125 μg/ml and 250 μg/ml were used. The antimycobactericidal capacity of the alkaloid extract from *C. zeyheri* was highly time-dependent. At the highest concentration of 250 μg/ml 100% bactericidal activity was achieved after a time period of 8 h. No growth was observed after 24 h. At 250 μg/ml the bactericidal activity of the extract was quite efficient and time-dependent with a decrease in the number of colony forming units per ml (cfu/ml) over time intervals 0, 2, 4, 6, 8 and 24 h respectively. At a concentration of 125 μg/ml a decrease in the number of cfu/ml was observed. The antimycobactericidal capacity was moderate with ≥ 90% killing achieved after 8 h calculated as the percentage reduction [27]. For both the 125 and 250 μg/ml, there was a decrease in the colony forming units which was time-dependent. In terms of efficiency of killing, the graph shows that the 250 μg/ml was more effective than 125 μg/ml at 0 and 4 h post incubation. However, in both cases of the concentrations, 100% killing activity was not achieved although concentration-dependent killing was achieved (Fig. 6). At 63 μg/ml, similar growth with the control was obtained. After 24 h *M. smegmatis* suspension in the 63 μg/ml sample was aggregated as characterised by the cloudy appearance of cells and this was probably due to increased mycobacterium cell density. Alkaloid extracts of *C. zeyheri* showed concentration-dependent killing with a decrease in the number of surviving colonies being observed from the lowest to the highest concentrations. A significant decrease in population of test organisms was observed at each interval with the percentage reduction in viable cell count observed to be from 40% to 90% and 13.3% to 100% between 2 to 24 h of interaction at the 125 μg/ml concentration and at 250 μg/ml concentration respectively. This represented a significant decrease in microbial population particularly at 250 μg/ml.

### Effect of plant extracts on Mycobacterium smegmatis efflux pumps

**Fig. 7:**
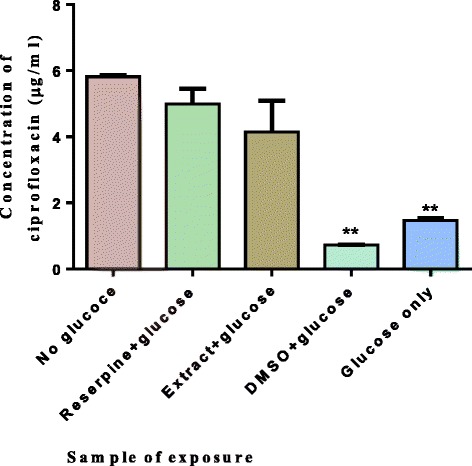
Effects of alkaloid extracts from *C. zeyheri* on drug accumulation in mycobacteria. Cells were loaded with ciprofloxacin and incubated for an hour. Cells were centrifuged, supernatant removed and exposed to the various compounds in buffer with glucose. One sample was exposed to the buffer alone with no glucose being added and this was the control. Cells were then centrifuged again and lysed with glycine-HCl. Cell debris was collected by centrifugation and the amount of ciprofloxacin in the supernatant quantified at excitation and emission wavelengths of 270 nm and 452 nm respectively using an RF1501 Shimadzu spectrofluorimeter. Values are the mean of ± SD for *n* = 2 for duplicate determinations **P < 0.01 in comparison with the cells exposed to glucose only

Of the 4 *Combretaceae* plant species tested; alkaloid extracts from *Combretum zeyheri* were the most effective in inhibiting drug efflux. The effect of extracts from *C. zeyheri* on efflux pumps in *M. smegmatis* was determined using the ciprofloxacin accumulation assay. Active accumulation of ciprofloxacin in *M. smegmatis* was monitored in the presence and absence of plant extracts and for comparison accumulation of ciprofloxacin was monitored in the presence of reserpine, a known efflux pump inhibitor. The ability of the plant alkaloid extracts in inhibiting efflux pump activity in *M. smegmatis* was comparable to that of reserpine a known efflux pump inhibitor. As shown in Fig. 7, there was no significant difference between the sample containing reserpine and glucose versus the sample containing the extract from *C. zeyheri* and glucose indicating that reserpine inhibited drug efflux. Similarly the alkaloid extract inhibited the efflux of ciprofloxacin from the cells to the same extent as reserpine.

## Discussion

Due to the emergence of multi-drug resistant mycobacterium, new alternatives for the treatment of tuberculosis are urgently needed. Statistics show that every year, 8 million people are infected with TB whilst 2 million deaths are reported annually [28]. When a patient becomes resistant to anti-tuberculosis drugs, particularly isoniazid and rifampicin, they have a 40% chance of survival regardless of their HIV status [6]. In addition, TB treatment protocols are long in duration causing patients to default from taking their medication and there is no sufficient treatment for multi-drug resistant TB (MDR-TB) [29]. At least 24 species of Combretum are well known in African traditional medicine and are used for the treatment of a variety of ailments and diseases, ranging from scorpion stings and snake bites, mental problems, heart and worm remedies and microbial infections [30]. The therapeutic effects of these medicinal plants is due to the presence of particular substances in plants most of which are phenols or their derivatives. These phytochemicals may not have direct physiological activity within the plant itself but have significant biological effects on animals [4].

Differences in yields for the four *Combretum* species were observed on extraction of the alkaloid phytoconstituents. This is not unusual as variations in biological activity and quantity of the phytochemicals present in the plant extract occur due to various reasons that include: plant age, differences in geographical areas in harvest sites and also seasonal variations [31].

When patients are inadequately exposed to anti-tuberculosis drugs, this may result in the emergence of resistant mycobacterium [31]. Drug resistance may be classified as phenotypic or genotypic resistance. Phenotypic drug resistance occurs when mycobacterium are only slightly affected by the anti-tuberculosis drugs such as rifampicin. This type of resistance maybe due to porin loss, increased efflux pump activity or presence of drug–modifying enzymes which will reduce desired drug concentrations intracellularly. Genotypic drug resistance is usually linked to mutations in mycobacterium genome and most of these mutations are known [9].

The uses of traditional medicines as alternative forms of health care provision are accepted, particularly in developing countries [32]. This led us to the investigation of the effects of alkaloid extracts from four *Combretaceae* family plant species on anti-mycobacterium activity. The need to use plant species belonging to the Combretaceae family of plants and investigating their effects on *M. smegmatis* was based on the ethnomedicinal use of these plants in Zimbabwe [33, 34] and other African countries in this region where the plants are used in the treatment of symptoms associated with TB such as persistent cough, chest pains and fever [8].

Using the broth microdilution method it was shown that alkaloid extract from *Combretum zeyheri* was the most potent against the mycobacterium with a minimum inhibitory concentration of 125 μg/ml and a minimum bactericidal concentration of 250 μg/ml. Extracts from *C. molle* and *C. platypetalum* were inactive with MICs above 1000 μg/ml whilst extracts from *C. apiculatum* were inconclusive. The criteria for an active plant extract has been given as MIC values of 128 μg/ml or IC_50_ values of less than 100 μg/ml [35]. Activity with plant extracts is considered to be moderate when 100 < MIC < 625 μg/ml [28]. In this regard, the alkaloid extract of *C. zeyheri* can be considered to have significant antimycobacterial effects. An MIC above 1000 μg/ml for crude extracts is categorised as being inactive against mycobacterium. This is because these are crude extracts of the plants and so MICs will not be a reliable indicator of the isolation of a potent antimycobacterial compound, thus, the lower the MIC, the more significant the result [36]. The front-line anti-TB drugs, such as rifampicin have MICs in the range of 0.025 – 0.20 μg/ml and if compared to the activity of any new extracts, a new compound must have an activity in the same range. However, these are crude extracts on which no further purification process has been carried out, hence, they may be considered active at higher concentrations as well particularly for *C. zeyheri*.

A time-kill assay aims to determine not only concentration-dependent killing capacity of drugs or plant extracts, but also the time – dependent killing activity of these agents. A wide range of concentrations of the drug or extract are used and mycobacterial killing at different time points is determined. This is done during exposure of the mycobacterium to the drug or plant extract being used. Therefore, instead of only obtaining results at the end – point; more detailed information is provided as to the different killing capacities of these agents at fluctuating concentrations [9]. This study, unlike a MIC/MBC assay, allows the determination of the speed of bactericidal activity of the extract [27].

An ideal anti-TB should show a high killing rate with a concomitant decrease in mycobacterial load reducing the risk of developing resistance or spreading the disease. The results of the present study provide insight into the in vitro killing dynamics of plant species belonging to the *Combretaceae* family of plants with respect to the killing capacity (concentration-dependence) and the rate of killing (time-dependence). Of interest is the adaptation or acclimatisation phase of microorganisms which was observed between the 2 h and 4 h interval at 125 μg/ml. This phase is characterised by some form of growth though minimal as microorganisms are acclimatising to the environment. However, because microbial cells were in a toxic environment, cell death occurred soon after and this was quite rapid at 250 μg/ml. A sharp decrease in microbial population was to be expected at higher concentrations (500 – 1000 μg/ml). However, the nature of the assay required concentration values close to those of the MIC of the most potent plant species.

Resistance of mycobacterium to anti-TB agents is hinged on a number of factors which include presence of efflux pumps which the major factor is contributing to intrinsic resistance [37]. Also, the nature of the mycobacterium cell wall makes it extremely impermeable to a wide range of agents due to its complex system of mycolic acids intercalated to a whole range of lipids and polymers [38].

Glucose transport systems in *M. smegmatis* belong to a family of transporters called the ATP-binding cassette (ABC) transporter family. *Mycobacterium tuberculosis* possesses five sugar import systems. However, *Mycobacterium smegmatis* has 28 such transporters which it also uses to pump out foreign compounds [39]. *M. smegmatis* is the microbial species of choice to use in learning more about mycobacterium physiology in vitro [21]. Furthermore, *M. smegmatis* is non-pathogenic in nature and is also fast growing [40]. Ciprofloxacin is a potent antimycobacterial agent (second line) that targets genetic material specifically DNA gyrase and topoisomerase IV. In order to study the transport of agents across cell membranes a detection compound must be utilized. Detection compounds vary and include use of radioactive isotopes of common efflux pump substrates [37]. In this study, ciprofloxacin was used to quantify the accumulated amount inside the cell, thereby, assessing the effects of plant extracts on the drug pumping activity of ATP-binding cassette proteins in *M. smegmatis*.

Plant alkaloid extracts from *C. zeyheri* were the most potent of the four plant extracts in inhibiting the transport of ciproflocaxin. Reserpine is a plant alkaloid-based drug which was used clinically as an antihypertensive agent and as an anti-psychotic agent as well, although rarely used nowadays clinically due to toxic adverse effects [41]. It is a known efflux pump inhibitor. Reduced levels of ciprofloxacin were pumped out of the mycobacterial cells compared to the cells incubated with glucose and hence, more of the drug accumulated within the cell. Though comparable with the activity observed with reserpine, the extract did not result in greater accumulation of the drug inside the cells. Considering the moderate MIC of extracts from *C. zeyheri* (125 μg/ml), and comparable efflux pump inhibition to a known inhibitor, reserpine, the results suggest that besides possessing antimycobacterial activity, inhibition of efflux pumps could be a mechanism of action of these extracts [42].

There are many mechanisms by which phytochemicals may act on Mycobacteria and these include inhibition of cell wall synthesis, protein synthesis, interference with membrane integrity and others [43]. The aim of this study was first determine if an alkaloid extract had an effect on the growth of *M. smegmatis*. Since Mycobacteria are known to be inherently resistant to antimycobacterial agents because of the complex cell wall, and also because they pump out xenobiotics including anti-TB drugs, we tested if the antimycobacterial action of the alkaloid extracts could be due to interference with drug efflux. Our results show that the alkaloid extract prevented the efflux of ciprofloxacin from *M. smegmatis*. These findings would assist in the isolation of alkaloid compounds that have efflux inhibitory activity in Mycobacteria from this plant. There are reports of increased efflux pump expression in *Mycobacterium tuberculosis* in clinical isolates and efflux pump inhibitors such as verapamil have been used to enhance the antimycobacterial effects [44, 45]. Thus, inhibition of efflux pumps should be manipulated as a target for new antimicrobial agents in drug development. Furthermore, if such promising antimicrobial effects are possessed with crude extracts that have not undergone an isolation and purification process then the extraction of the active compound responsible for these effects might result in greater inhibitory activity.

## Conclusions

The alkaloid extracts from the plant species, *C. zeyheri*, were the most potent and displayed potent antimycobacterial activity towards *M. smegmatis*. In addition not only was there concentration-dependent killing capacity observed but also, time time-dependent killing activity as well. The extracts showed potential efflux-pump inhibitory activity, hence, further studies of this plant may lead to isolation of the bioactive compound. It is, therefore, essential to carry out isolation, purification and antimycobacterial evaluation of phytochemicals from *Combretum zeyheri*.

## Abbreviations

ABC: ATP binding Cassette proteins
cfu: Colony forming units
DMSO: Dimethyl sulphoxide
DNPs: Dictionary of Natural Products
INH: Isoniazid
MBC: Minimum bactericidal concetrations
MDR: TB-Multidrug resistant tuberculosis
MIC: Minimum inhibitory concentration
MTT: 3-(4, 5-dimethylthiazolyl)-2, 5-diphenyltetrazolium bromide
RIF: Rifampin
TB: Tuberculosis
